# The High Prevalence of Functional Complement Defects Induced by Chemotherapy

**DOI:** 10.3389/fimmu.2016.00420

**Published:** 2016-10-17

**Authors:** Mischa P. Keizer, Angela M. Kamp, Cathelijn Aarts, Judy Geisler, Huib N. Caron, Marianne D. van de Wetering, Diana Wouters, Taco W. Kuijpers

**Affiliations:** ^1^Sanquin Research and Landsteiner Laboratory AMC, Department of Immunopathology, University of Amsterdam, Amsterdam, Netherlands; ^2^Academic Medical Center (AMC), Emma Children’s Hospital, University of Amsterdam, Amsterdam, Netherlands; ^3^Sanquin Research and Landsteiner Laboratory AMC, Department of Blood Cell Research, University of Amsterdam, Amsterdam, Netherlands

**Keywords:** complement system, transient defects, oncology, therapeutic effect, methotrexate

## Abstract

**Introduction:**

To date, oncology patients are more dependent on non-cellular host defense against pathogens due to intensive (chemo)therapy-related bone marrow suppression. Since data on complement functionality in oncology patients are limited, we aimed to investigate the innate complement function in relation to the type of malignancy and therapy in a longitudinal cohort of patients.

**Methods:**

A large single-center, prospective non-intervention study was conducted, in which blood samples were taken from patients before, during, and after treatment with chemotherapy and/or subsequent admittance for (febrile) neutropenia.

**Results/findings:**

Analysis of 48 patients showed a high percentage of defects in complement activity of the alternative pathway (19.1%), the classical pathway (4.3%), or both (42.6%). *Post hoc* analysis of six different treatment protocols with more than three patients each showed distinct effects of specific therapies. Whereas patients treated according to the Ewing, EpSSG-rhabdomyosarcoma, or SIOP CNS germ cell tumor protocol showed no defects, patients treated according to the ALL-11 (leukemia), the EURAMOS I (osteosarcoma), or the ACNS (medulloblastoma) protocols showed an almost universal reduction in complement function. Although we could not explain the reduced complement functionality under all conditions, a strong effect was observed following high-dose methotrexate or ifosfamide.

**Conclusion:**

Acquired complement defects were commonly observed in more than 50% of oncology patients, some of which associated with certain chemotherapeutic drugs. Additional studies are needed to determine the clinical and therapeutic context of complement defects and their possible effect on treatment outcome or the increased risk of infection.

## Introduction

Infection is the most important cause of treatment-related deaths in oncology and the second most common reason for hospitalization during therapy (1, 2). The risk of infection and inflammation is increased in oncology patients due to treatment-induced neutropenia (3), making them more dependent on their innate, non-cellular immunity. This dependence on innate host defense mechanisms might be more prominent in children because of the relative immaturity of the immune system (4). Both the intactness of the barriers of skin, pulmonary, and gastrointestinal surface membranes as well as a functional innate immunity provide the most relevant defense against bacterial and fungal pathogens in oncology patients. To a large extent, this depends on complement proteins recognizing conserved so-called pathogen-associated molecular patterns (PAMPs) on the microbes on these surfaces upon invasion when these barriers are breached, for instance, by chemotherapy or radiation effects.

Although complement components are synthesized early in gestation, neonates show an immature innate immune response at birth, which poses an increased risk for infection. Concentrations of complement components are lower early during infancy compared to adults but rapidly normalize within weeks after birth (5, 6). Complement activation leads to opsonization of pathogens for phagocytosis, induces an inflammatory response, and eventually lyses pathogens *via* the membrane attack complex. Activation is induced *via* three distinctive pathways, the classical pathway (CP), the lectin pathway (LP), and the alternative pathway (AP) (7). Binding of antibody to antigen can activate the C1 complex (composed of C1q, C1r, and C1s), which will lead to the activation of C4 and C2, resulting in the generation of a C3-convertase C4b2a to activate the terminal pathway (8). In the LP, the binding of mannan-binding lectin (MBL), or one of the different ficolins (FCN), to their specific ligands will activate the MBL-associated serine proteases (MASPs), hereby generating a C3-convertase *via* a similar mechanism to the CP (9). Activation of the AP can occur *via* the spontaneous activation of C3 to C3(H_2_O), which, if not inhibited, generates a C3-convertase [C3(H_2_O)Bb], or *via* activation on apoptotic cells (10), or *via* amplification of the initial response of the CP or LP (11). The formation of a C3-convertase activates the terminal pathway, resulting in the formation of the membrane attack complex and lysis of the target cell (7, 12).

Although a role for complement factors has been described for the host immunosurveillance against cancer (13–15), recent papers have instead confirmed a potentially harmful role of complement *via* the active C3a and C5a fragments that may contribute to the initiation of malignant cells (16, 17). Limited data have been published regarding the effect of cancer therapeutics on complement components, and either way, both the effect of therapy on the protein levels of complement components (18) or the effect of therapy on the functionality of the complement pathways have not been well studied to date (19, 20).

Our previous report on a small cohort of oncology patients during MBL-substitution therapy suggested preexisting defects in complement activation (19). To confirm these previously observed reductions in complement activation in oncology patients and to correlate the effect of chemotherapy on the complement system in greater detail, a longitudinal observational study was conducted in patients being treated for diverse malignancies, including hematological malignancies and various solid tumors.

## Materials and Methods

### Study Design and Protocol

Between 1 September, 2012 and 1 September, 2014, all pediatric oncology patients who were admitted to the oncology department of the Emma Children’s Hospital, Academic Medical Center (AMC), Amsterdam, the Netherlands, were eligible for inclusion in the complement study (C2012). After inclusion, patients were anonymized after written informed consent from parents and children (>12 years) was obtained. The study was conducted according to the declaration of Helsinki and Good Clinical Practice. The study protocol was approved by the local ethics committee (CCMO registered NL39747.018.12).

### Patient Selection

**Figure 1:**
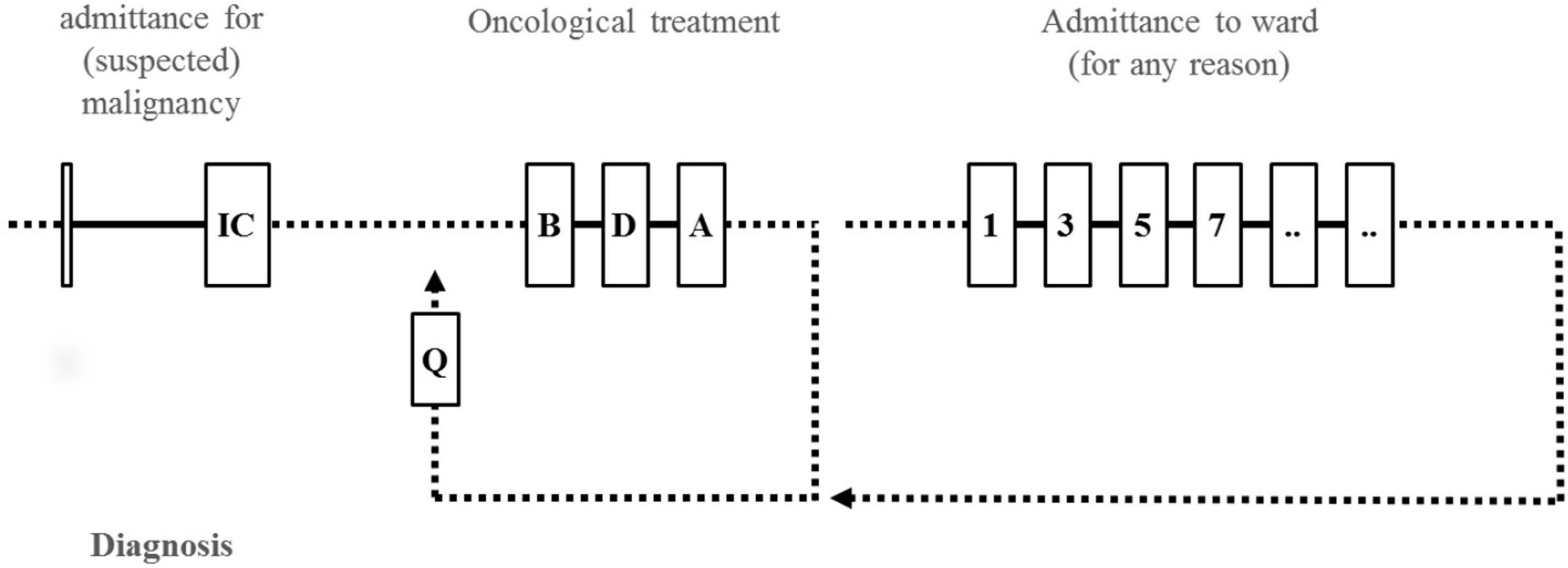
**Design of the complement study**. (When possible) blood was obtained during diagnosis (*T* = IC). Further blood sampling of included patients took place during oncological treatment [before start (*T* = B), during (*T* = D), or after treatment (*T* = A)] or during admittance for any other reason, such as, but not limited to (suspected) infection or episodes of febrile neutropenia (FN). Serial blood samples were drawn every other day (*T* = 1, *T* = 3, *T* = 5, etc.) during admittance. After admittance for therapy or episodes of FN, patients were sampled when qualifying (*T* = Q) for therapy.

Eligibility criteria included pediatric oncology patients, irrespective of the treatment of the tumor (e.g., chemotherapy, surgery, radiotherapy, or combinations hereof), who were admitted to the hospital for oncological treatment or admitted to the oncological ward for any other reason, such as, but not limited to, suspected or proven infection (Figure 1). Blood was drawn at specific time points (*T*), when possible, on the day of diagnosis (*T* = IC), before the start of therapy (*T* = B), during therapy (*T* = D), and after therapy (*T* = A), and thereafter, during episodes of febrile neutropenia (FN; neutropenia <500 cells/μL) or periods of suspected infection, continuing during admission with blood serial sampling every other day (*T* = 1, *T* = 2, etc.), only at moments of routine blood sampling for any clinical indication. The next time point was when the patient was recovered from FN (*T* = Q, recovery of FN) or proven infection.

### End-Points

The primary end-points of the study were (1) the presence and prevalence of transient reduced complement functionality, (2) determining the effect of therapy and malignancy to reduced functionality of complement activation, i.e., the association of reduced complement functionality with specific therapeutic regimes or specific malignancies, if possible, and (3) determining the association of neutropenia and reduced complement functionality. Data on the occurrence and duration of fever, (proven) infection, and medication were obtained from the patient files. To determine a possible effect of therapy, only the largest groups of patients with similar treatment were selected for further *post hoc* analysis. Observational periods were defined as the period of days between admittance to the hospital, often related to oncological treatment or FN, and time of discharge. Because characteristics related to therapy and course of disease could change over time, different observation periods per patient could be defined. Diagnostic results (neutropenia, cell counts, organ failure) and different clinical parameters (mucositis, general well-being) were analyzed in relation to complement function. Total levels of complement component C3 (gram per Liter) and the complement C3 activation-dependent split product C3d [compared to fully activated normal serum aged (NSA) for 1 week at room temperature] were both obtained from the diagnostic department.

### Power Analysis

Transient reduction of functionality of the AP of complement was first observed during an MBL-substitution study in MBL-deficient patients. The number of patients required to determine a transient reduction of complement functionality was calculated at 24 patients. Although the activation of the AP is independent of the activation of the LP or CP, this clinical observational study was corrected for the distribution of MBL-deficiency within the normal population.

### Blood Samples

Blood samples from the patients were obtained during routine sampling. All blood samples were processed within 1 h, and serum, plasma, and buffy coat were isolated, according to the previously described protocols (19, 21), and stored at −80°C until use.

### Assays

Functionality of the three complement activation pathways was performed at Sanquin Research and the Landsteiner laboratory, AMC, Amsterdam, using the well-described commercially available Wielisa^®^ Complement system Screen COMPL 300 (Wieslab, Lund, Sweden) (22, 23). The ELISA was developed according to the manufacturer’s instructions. Briefly, specific pre-coated plates for the different complement activation pathways were incubated with a fixed dilution of serum, depending on the pathway of interest, and compared to a positive control (%PC), which is given the value 100%.

Due to the strong influence of MBL levels on the functionality of the LP (24), both MBL serum levels and MBL genotype were determined to confirm the results of the Wielisa^®^. MBL genotyping was performed by Taqman assay, as previously described (25). By random selection of five samples per patient (if a series of more was available), MBL serum levels were measured as described before (19). Briefly, after incubation of serum to mannan-coated plates, mannan-bound MBL was detected using biotinylated mouse-anti MBL (αMBL-1) (26) and compared to a serum pool (with a known concentration of 1.3 μg/mL).

The AP functionality was also measured by AP50 (i.e., the amount of serum needed for 50% hemolysis of rabbit erythrocytes by AP activation) (27). By incubating lower dilutions of serum with rabbit erythrocytes in the presence of calcium chelators to prevent CP and LP activation, the functionality of the AP can be selectively assessed. Activation of the AP will result in lysis of the rabbit erythrocytes, and the increase in absorbance of released hemoglobin can be measured in the cell-free supernatant as compared to a 100% lysis control obtained by incubation of the erythrocytes with saponin [reference values: 75–125% (28)].

### Statistical Analysis

The association between MBL levels and functionality was calculated using a one-phase association with ordinary fit (no restriction). To determine a temporal effect of therapy on the functionality of the complement system, patient samples were paired within the different chemotherapy sessions. Significance was calculated with a paired, two-sided *T*-test. Data are expressed as mean ± SD in case of normal distributed data, unless otherwise mentioned, and as median (range) for not normally distributed data. *P-*values less than 0.05 were considered statistically significant.

## Results

### Baseline Characteristics

Patient characteristics are described in Table 1. The total number of patients included in C2012 was 48. Patients were coded CA-001 to CA-048. The median age of the patients [27 males (56.3%), 21 females (43.7%)] was 8.0 years (range: 0.3–17.6). The median follow-up was 488.5 days (range: 49–727), for a total number of 794 time points. Each patient received a unique identification number and was followed during different admittances to the hospital. One patient (CA-017) was included but was never admitted to the hospital and therefore excluded from further analysis. The larger diagnostic groups analyzed were acute lymphoblastic leukemia (ALL) (*n* = 5; 10.4%), osteosarcoma (*n* = 7; 14.6%), Ewing sarcoma (*n* = 5; 10.4%), rhabdomyosarcoma (*n* = 3; 6.3%), germ cell tumors (*n* = 3; 6.3%), and medulloblastoma (*n* = 3; 6.3%). Detailed description of functionality of the complement system of the specific diagnostic groups is described in Table 2. The remainder of the patients (*n* = 21; 43.8%) was not analyzed for specific associations with therapy or malignancy, due to the low number of similar therapies or lack of shared diagnoses within the study population.

**Table 1:** **Patient characteristics**.

Patient	Sex^a^	Age (years)	Tumor			Therapy protocol		Follow-up (days)	T^e^	MBL genotype
				S^b^	G^c^	R^d^				
CA-001	M	3.2	Ewing sarcoma	S	VIIIc		EuroEwing	197	4	LXPA/LXPA^f^
CA-002	M	1.9	AML	H	Ib		DB AML 01	727	8	HYPA/HYPA
CA-003	M	3.3	Neuroblastoma	S	IVa		HR chemo	713	19	HYPA/LXPA
CA-004	F	3.4	Pre B ALL	H	Ia		ALL-11	684	77	HYPA/HYPA^f^
CA-005	M	17.6	BNHL	H	IIb		LMB 2001 C	684	14	HYPA/LXPA
CA-006	F	11.7	GCT	S	X		MAKEI 96	150	3	LYPA/LYQA
CA-007	F	6.2	Pre B ALL	H	Ia		ALL-11	676	43	HYPA/LYQA^f^
CA-008	M	17.0	GCT	S	X		SIOP CNS GCT 2009	669	5	LYQA/LYQC^f^
CA-009	F	2.5	Pleuropulmonal blastoma	S	XIIa	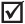	Chemotherapy	656	17	HYPD/LXPA
CA-010	M	9.9	GCT	S	X		SIOP CNS GCT 2009	655	3	LXPA/LYQA^f^
CA-011	M	15.6	Ewing sarcoma	S	VIIIc		Ewing 2008	643	29	LYPB/LYQA^f^
CA-012	F	15.8	Osteosarcoma	S	VIIIa	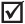	EURAMOS I	641	28	LYPB/LYQA^f^
CA-013	M	14.9	Burkitt lymphoma	H	IIc		COP COPADM1	630	7	HYPA/LYPB
CA-014	M	12.0	Hodgkin	H	IIa		Euronet PHL CL1	616	9	HYPA/LYPB
CA-015	F	14.7	Osteosarcoma	S	VIIIa	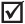	EURAMOS I	599	28	HYPA/LYPA^f^
CA-016	M	3.9	Alveolar RMS	S	IXa		EpSSG-RMS 2005	598	24	HYPA/LYPA^f^
CA-017	M	1.5	Neuroblastoma	S	IVa	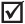	Chemotherapy	588	0	n/a
CA-018	M	10.8	Burkitt lymphoma	H	IIc		LMB 2001	588	23	HYPA/HYPA
CA-019	F	1.6	Ependymoma	S	IIIa	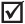	Chemotherapy	587	42	LXPA/LXPA
CA-020	M	16.3	ALL	H	Ia		ALL-11	574	32	HYPA/LXPA^f^
CA-021	M	14.4	Ewing sarcoma	S	VIIIc		Ewing 2008	567	12	LXPA/LYQA^f^
CA-022	M	12.8	Osteosarcoma	S	VIIIa		EURAMOS I	329	17	LYPB/LXPA^f^
CA-023	M	6.5	T-ALL	H	Ia		ALL-11	549	27	HYPD/HYPA^f^
CA-024	F	0.3	Infant ALL	H	Ia		Interfant 06 HR	49	8	LXPA/LYQC
CA-025	M	12.7	GCT	S	X		SIOP CNS GCT 96	532	12	LYPB/LYQC^f^
CA-026	M	2.9	Melanotic neuroectodermal tumor	S	VIIIc	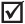	Chemotherapy	522	9	LYPA/LYQA
CA-027	M	1.7	Opticus glioma	S	IIIc		SIOP-LGG 2004	507	14	HYPA/LXPA
CA-028	F	14.9	Anaplastic large-cell lymphoma	H	II		ALCL-99	490	14	HYPA/HYPD
CA-029	M	14.5	Hodgkin lymphoma	H	IIa		Euronet PHL-C1 TG3	487	10	HYPA/LYPB
CA-030	M	14.5	Osteosarcoma	S	VIIIa	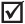	EURAMOS I	423	33	LYPB/LYQA^f^
CA-031	F	4.3	Atypical teratoid rhabdoid tumor	S	IIIc		Euro rhabdoid CNS 2010	405	19	LXPA/LYQA
CA-032	M	2.9	RMS D	S	IXa		EpSSG-RMS 2005 SR	289	6	HYPD/LYQA^f^
CA-033	F	7.7	Osteosarcoma	S	VIIIa	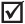	EURAMOS I	375	25	LYQA/LXPA^f^
CA-034	F	7.5	Ewing sarcoma	S	VIIIc		Ewing 2008	371	13	LYQC/LYPA^f^
CA-035	F	2.4	PNET	S	IIIc	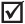	HEADSTART III	371	12	HYPD/HYPA
CA-036	F	14.7	Osteosarcoma	S	VIIIa	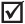	EURAMOS I	367	13	HYPA/LYQA^f^
CA-037	M	13.8	Osteosarcoma	S	VIIIa	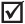	EURAMOS I	333	33	HYPA/LXPA^f^
CA-038	F	12.5	Medulloblastoma	S	IIIc		ACNS0331	344	8	HYPD/HYPA^f^
CA-039	M	6.9	Medulloblastoma	S	IIIc		ACNS0332	313	9	HYPA/LYPB^f^
CA-040	F	8.2	Medulloblastoma	S	IIIc		ACNS0332	307	14	LXPA/LYQA^f^
CA-041	M	13.2	CNS germinoma	S	Xa		CNS GCT 2009	293	2	n/a^f^
CA-042	F	16.4	B-NHL	H	IIb		B-NHL Skion	258	12	LYPB/LYQA
CA-043	F	1.8	ALL	H	Ia		ALL-11	213	22	LXPA/LYQA^f^
CA-044	F	13.2	Osteosarcoma	S	VIIIa	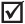	EURAMOS I	60	12	HYPA/LYPB^f^
CA-045	M	0.7	Embryonal RMS	S	IXa		EPSSG subgroup c	188	5	HYPA/LXPA^f^
CA-046	F	1.8	Ewing sarcoma	S	VIIIc		EWING 2008	160	4	LXPA/LXPA^f^
CA-047	M	2.9	Hepatoblastoma	S	VII		SIOPEL 3 SR	107	6	LXPA/LXPA
CA-048	F	5.3	Wilms tumor	S	VIa		SIOP 2001	97	8	HYPA/HYPA

*^a^F(emale) or M(ale)*.

*^b^S(olid) or H(ematological) malignancy*.

*^c^Classification of diagnostic group of malignancy according to Steliarova-Foucher et al. (29)*.

*^d^R(esection)*.

*^e^T(imepoints)*.

*^f^Selected for further analysis*.

**Table 2:** **Complement activation functionality of patients within six different therapy protocols**.

Patient (***n*** = 5)	Age (years)	Tumor	Therapy	Follow-up (days)	Time points (*n* = 201)	CP		AP	
						Median% PC (range)		Median% PC (range)	
CA-004	3.4	Pre B ALL	ALL-11	684	76^a^	85.2	0–123.2	38.7	0–113.5
CA-007	6.2	Pre B ALL	ALL-11	676	43	91	11.1–109.8	77	0–112
CA-020	16.3	ALL	ALL-11	574	32	86.5	1–120	49.5	0–118
CA-023	6.5	T-ALL	ALL-11	549	27	80.5	2–124	17	0–135
CA-043	1.8	ALL	ALL-11	213	22	103.8	86.4–106.2	81.4	79.9–100
		Median (range)		574 (213–684)	32 (22–77)	87.3	(0–124)	48.7	(0–135)

**Patients (*n* = 7)**	**Age (years)**	**Tumor**	**Therapy**	**Follow-up (days)**	**Time points (*n* = 189)**	**CP**		**AP**	
						**Median% PC (range)**		**Median% PC (range)**	

CA-012	15.8	Osteosarcoma	EURAMOS I	641	28	100	84.7–117.3	100	68.6–113.5
CA-015	14.7	Osteosarcoma	EURAMOS I	599	28	96	14.4–116.4	70.5	2.1–100
CA-022	12.8	Osteosarcoma	EURAMOS I	329	17	97.2	67–110	94.2	2–116
CA-030	14.5	Osteosarcoma	EURAMOS I	423	33	91	53–121.2	81	4.9–115.4
CA-033	7.7	Osteosarcoma	EURAMOS I	375	25^a^	106	59.9–133.6	87.7	11.5–120.4
CA-036	14.7	Osteosarcoma	EURAMOS I	367	13^a^	101.5	33.3–134.7	79.8	32–105
CA-037	13.8	Osteosarcoma	EURAMOS I	333	33^a^	103.3	0–139.5	95.9	0–217.3
		Median (range)		375 (60–641)	25 (12–33)	100.0	(0–139.5)	87.60	(0–217.3)

**Patients (*n* = 5)**	**Age (years)**	**Tumor**	**Therapy**	**Follow-up (days)**	**Time points (*n* = 62)**	**CP**		**AP**	
						**Median% PC (range)**		**Median% PC (range)**	

CA-001	3.2	Ewing sarcoma	EuroEwing	197	4	100	100–100	98	83–100
CA-011	15.6	Ewing sarcoma	Ewing 2008	643	29	91	23.9–100.1	84.4	4.1–108
CA-021	14.4	Ewing sarcoma	Ewing 2008	567	12	95.25	67.1–106.8	100	72.78–131.3
CA-034	7.5	Ewing sarcoma	Ewing 2008	371	13	100	62.8–117.7	98.3	40.5–120.8
CA-046	1.8	Ewing sarcoma	Ewing 2008	107	4	99.5	92–128.6	101.3	92.3–105
		Median (range)		371 (107–643)	13 (4–29)	97.05	23.90–128.6	96.90	4.1–131.3

**Patients (*n* = 3)**	**Age (years)**	**Tumor**	**Therapy**	**Follow-up (days)**	**Time points (*n* = 35)**	**CP**		**AP**	
						**Median% PC (range)**		**Median% PC (range)**	

CA-016	3.9	Alveolar RMS	EpSSG-RMS 05	598	24	97.65	75.4–111.4	78.3	7.6–102
CA-032	2.9	RMS	EpSSG-RMS 05	289	6	93.05	32–112.7	62	1–86.2
CA-045	0.7	Embryonal RMS	EpSSG sgc	188	5	113.8	94.24–130.1	46.62	22.5–128.4
		Median (range)		289 (188–598)	6 (5–24)	99.10	32–130.1	66.83	1–128.4

**Patients (*n* = 3)**	**Age (years)**	**Tumor**	**Therapy**	**Follow-up (days)**	**Time points (*n* = 20)**	**CP**		**AP**	
						**Median% PC (range)**		**Median% PC (range)**	

CA-008	17.0	GCT	SIOP CNS GCT 2009	669	5	100	95.2–103.4	100	62.3–100
CA-010	9.9	GCT	SIOP CNS GCT 2009	655	3	100	98.3–100	96.3	73–100
CA-025	12.7	GCT	SIOP CNS GCT 1996	532	12	97.4	74–110.4	95	6.0–100
		Median (range)		655 (532–669)	5 (3–12)	99.6	74–110.4	97.1	6.0–100

**Patients (*n* = 5)**	**Age (years)**	**Tumor**	**Therapy**	**Follow-up (days)**	**Time points (*n* = 31)**	**CP**		**AP**	
						**(%PC) (range)**		**(%PC) (range)**	

CA-038	12.5	Medulloblastoma	ACNS0331	344	8	100.2	80.5–124.9	40.25	6.8–56.50
CA-039	6.9	Medulloblastoma	ACNS0332	313	9	95.40	75.5–123.1	29.00	4.0–100.0
CA-040	8.2	Medulloblastoma	ACNS0332	307	14	112.5	78.44–137	61.29	12.10–164.7
		Median (range)		307 (307–344)	9 (8–14)	106.1	75.5–137	44.50	4.0–164.7

*^a^Due the experimental and logistical error, not all samples could be measured*.

### The Lectin Pathway of Complement

One single complete MBL-deficiency was observed in a patient with MBL protein levels below the detection limit (CA-025). This deficiency was confirmed by genotyping. No other complete genetic complement deficiencies were found within the included patients, since all patients showed normal functionality of the different activating pathways of complement during the observational period at several independent time points.

**Figure 2:**
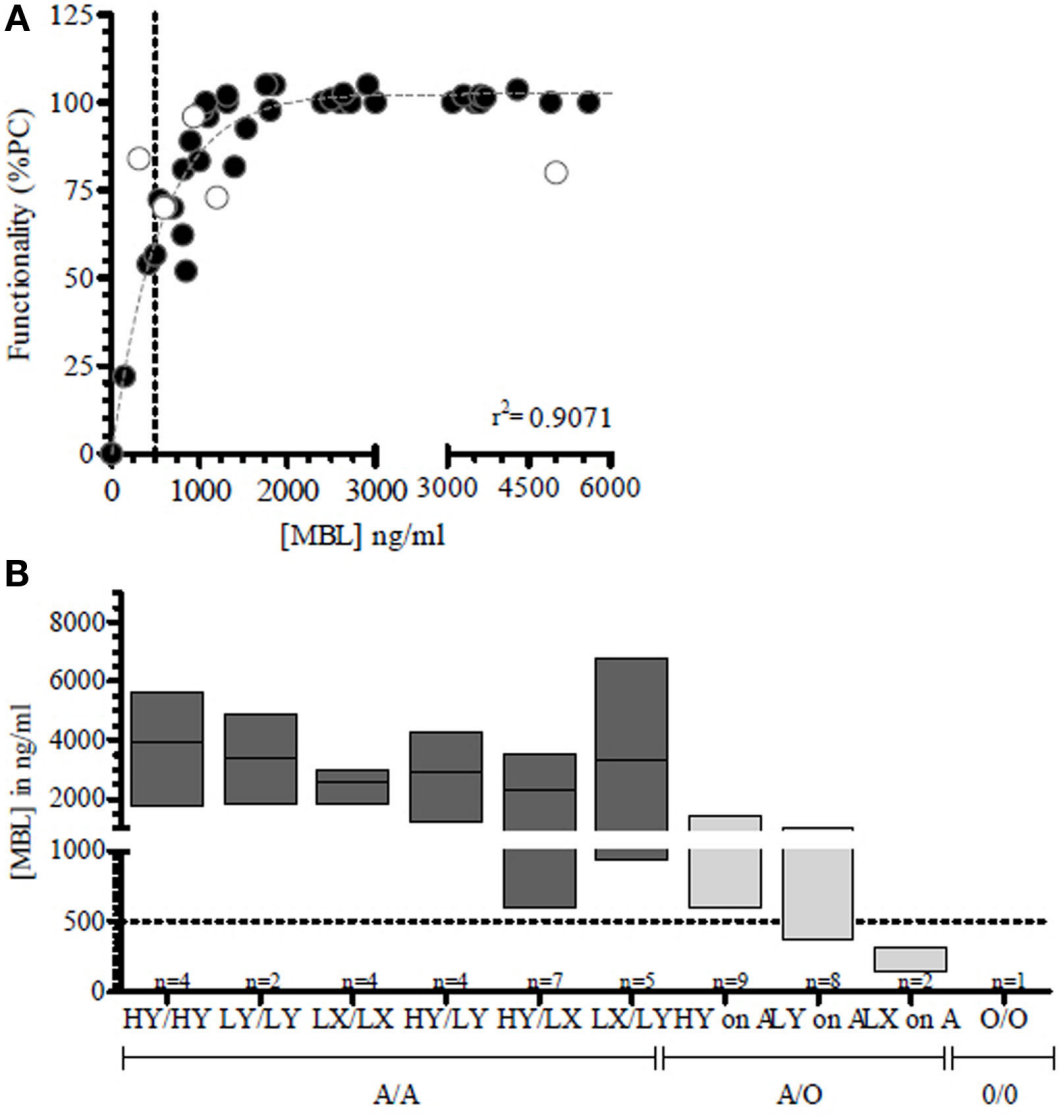
**Lectin pathway functionality correlation with MBL-concentration**. **(A)** In a longitudinal series of more than five consecutive time points per patient, MBL concentrations were measured in oncology patients (●) and correlated to the LP functionality (*r*^2^ = 0.907). Functionality is depicted as percentage of a positive control (%PC). **(B)** MBL genotype was correlated to MBL serum levels. MBL deficiency as defined as MBL levels below 500 ng/mL is depicted as a dotted line. Because of the observed effects of chemotherapy of the pediatric ALL-11 protocol (○) on the lectin pathway of complement, these ALL patients were excluded from the analysis (*n* = 5) (Keizer et al., submitted). MBL deficiency as defined by MBL levels below 500 ng/mL is depicted by a dotted line.

Activation of the LP of complement was directly related to MBL serum levels (*r*^2^ = 0.9071) (Figure 2A) and genotype (Figure 2B). Since MBL is a highly variable plasma protein based on genotype and acute phase response reactivity (30) (Appendix B in Supplementary Material), we focused in our subsequent analyses solely on the functionality of the CP and AP cascade of complement activation.

### The Classical and Alternative Pathway of Complement

**Figure 3:**
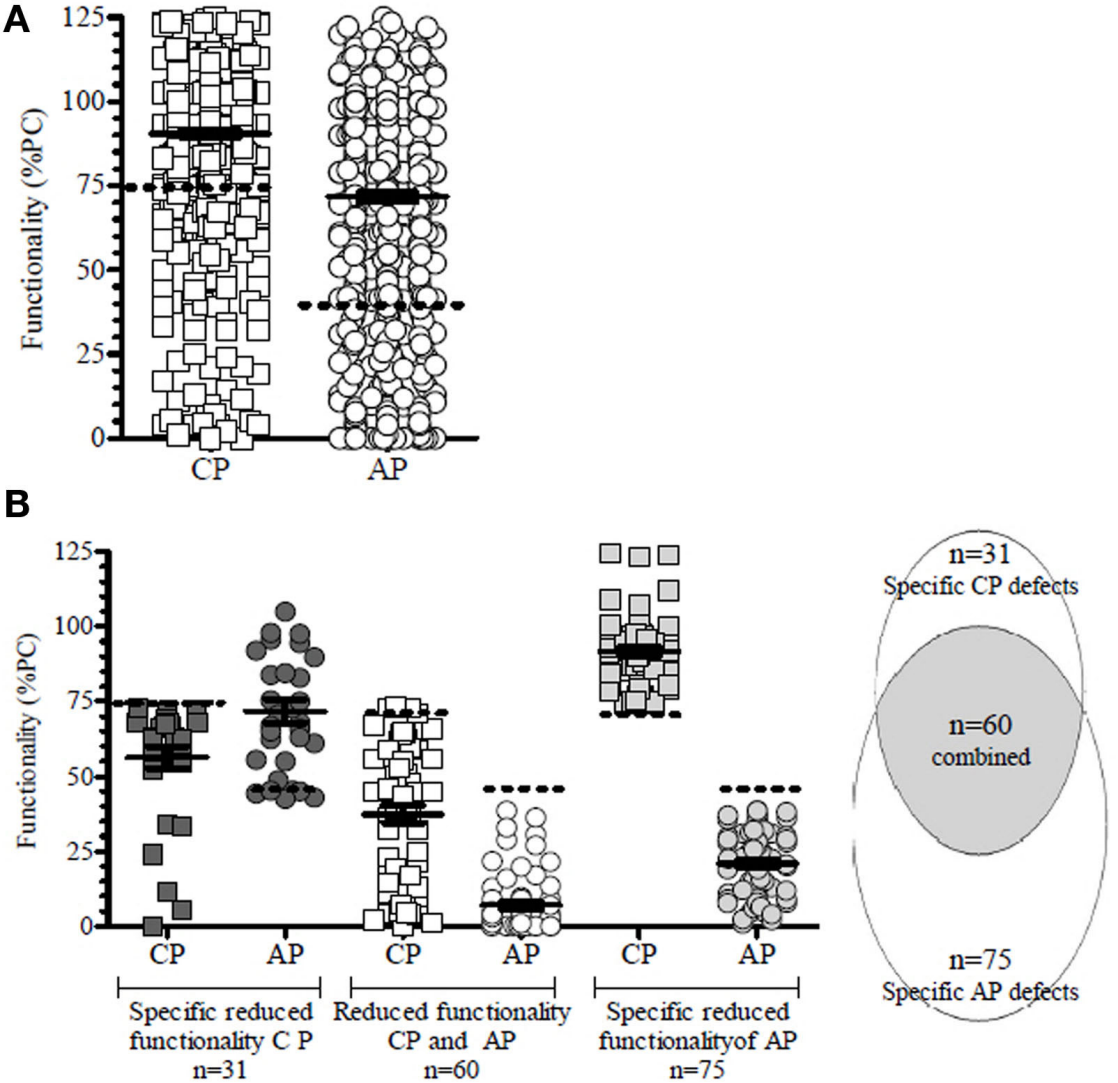
**Classical (CP) and alternative (AP) pathway complement activity at all time points in all patients of the C2012 study**. **(A)** In 13.5% of the time points showed a reduced functionality of the CP, and in 18.9% a reduced AP activity was observed. **(B)** Reduced functionality of the CP was divided into specific pathway inhibition (*n* = 31; 4.6%) or coincided with reduced AP activity (*n* = 60; 8.8%). Specific AP defects accounted for 11.5% (*n* = 75). The dotted line depicts the lower limit of functionality in controls, as defined by Seelen et al. (22) for CP functionality (abnormal <75%) and AP functionality (<40%) in the WIELISA assay. Functionality is depicted as percentage of a positive control (%PC).

The cutoff for abnormal functionality of the CP was measured by Wielisa^®^ COMPL 300 set at <75% and for the functionality of the AP at <40% of the positive control levels (%PC) (22). We observed a wide range of complement activities in our patients for the CP, from being completely inactive to normal [median 96% (range 0–137.0%)]. This was also true for the AP activity [median 79% (range 0–164.7%)] (Figure 3A).

No reduced functionality at any point was observed in 34.0% patients, whereas a reduced functionality was observed in both pathways in 42.6%, or in a single pathway in 23.4% (i.e., 4.3% in the CP and 19.1% in the AP).

In total, 24.8% (*n* = 166/669) of the time points analyzed showed a reduced functionality of the complement system in either the CP or the AP, or both (Figure 3A): specific CP defects were observed in 18.7% (*n* = 31/166) of all defects, whereas a reduction of the AP was seen in 45.2% (*n* = 75/166) of the time points (Figure 3B; Appendix C in Supplementary Material). A reduction of CP functionality coincided with a reduction of the functionality of the AP in 36.1% (*n* = 60/166) of time points with reduced functionality (Figure 3B). Since there was not a complete overlap, our findings indicated that selective CP or AP defects in oncology patients may occur. The number of patients in which these specific defects in CP (4.6% of all time points) and or AP (11.2% of all time points) do not coincide is suggestive for the presence of some as yet unknown factors, which have a selective deleterious impact on these complement pathways.

### Correlation of Complement Functionality to Specific Protocols

**Figure 4:**
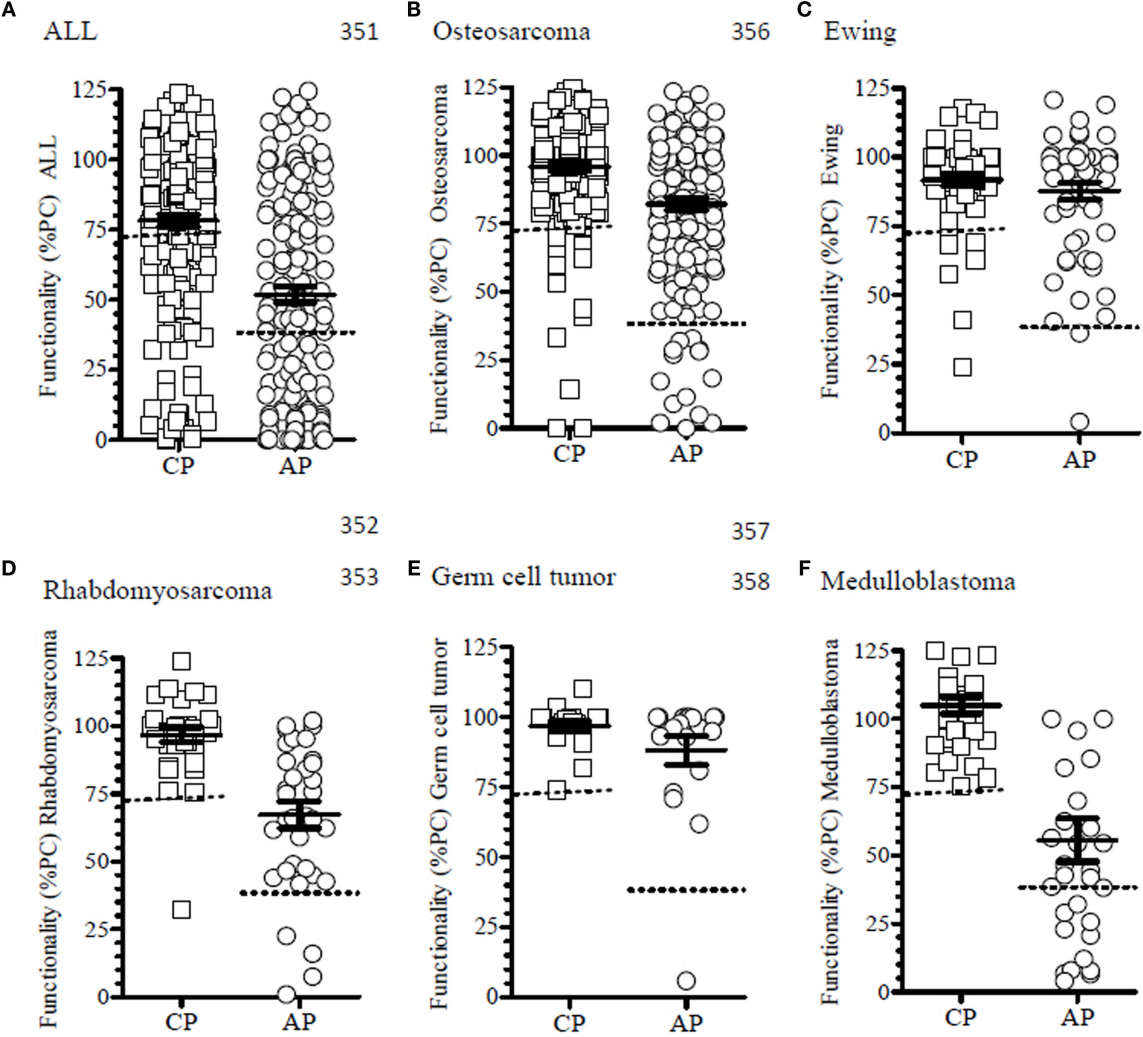
**The functionality of the complement system of patients within the different treatment protocols**. **(A)** In patients with ALL, 47.6% (*n* = 79/200) of the time points showed a reduced functionality of the complement system. There were 63.3% (*n* = 50/79) and 91.1% (*n* = 72/79), which were related to the CP or AP, respectively. **(B)** In patients with osteosarcoma and treated according to the EURAMOS I protocol, 23 time points (12.2%) had a reduction in complement functionality. Of these, 23 moments 60.9% (*n* = 14/23) and 78.3% (*n* = 18/23) where in CP and AP, respectively. **(C)** Ewing sarcoma patients had in 14.5% (*n* = 9/62) a reduced functionality, but they were mostly related to the CP (88.9%, *n* = 8/9) and to a lesser extent to the AP (22.2%, *n* = 2/9). **(D)** Patients with rhabdomyosarcoma treated according to EpSSG protocol showed in 11.4% of the analyzed samples complement defects (*n* = 4), which were all related to the AP (100%). **(E)** Patients with germ cell tumors had no reduced functionality apart from a single time point in AP activity (5%, *n* = 1). **(F)** Patients with medulloblastoma had only defects in 41.9% of the samples (*n* = 13/31) and all were in the AP. Mean ± SEM are shown. Functionality is depicted as percentage of a positive control (%PC).

To determine potential drug-induced inhibition on the complement system, the patients were analyzed in specific treatment-related groups, and functionality was analyzed in relation to the harmonized therapy protocols used to treat these patients. Due to the heterogeneity of the patient population, the top six therapy groups were selected for further analysis of some of these oncological patient subgroups (Table 2). All patients with ALL, patients with osteosarcoma (*n* = 6/7), and all patients with medulloblastoma showed reduced CP and/or AP functionality at several time points following chemotherapy, whereas patients with Ewing sarcoma or germ cell tumors had no significant reduction in complement activation upon treatment for their solid tumor (Figure 4).

**Figure 5:**
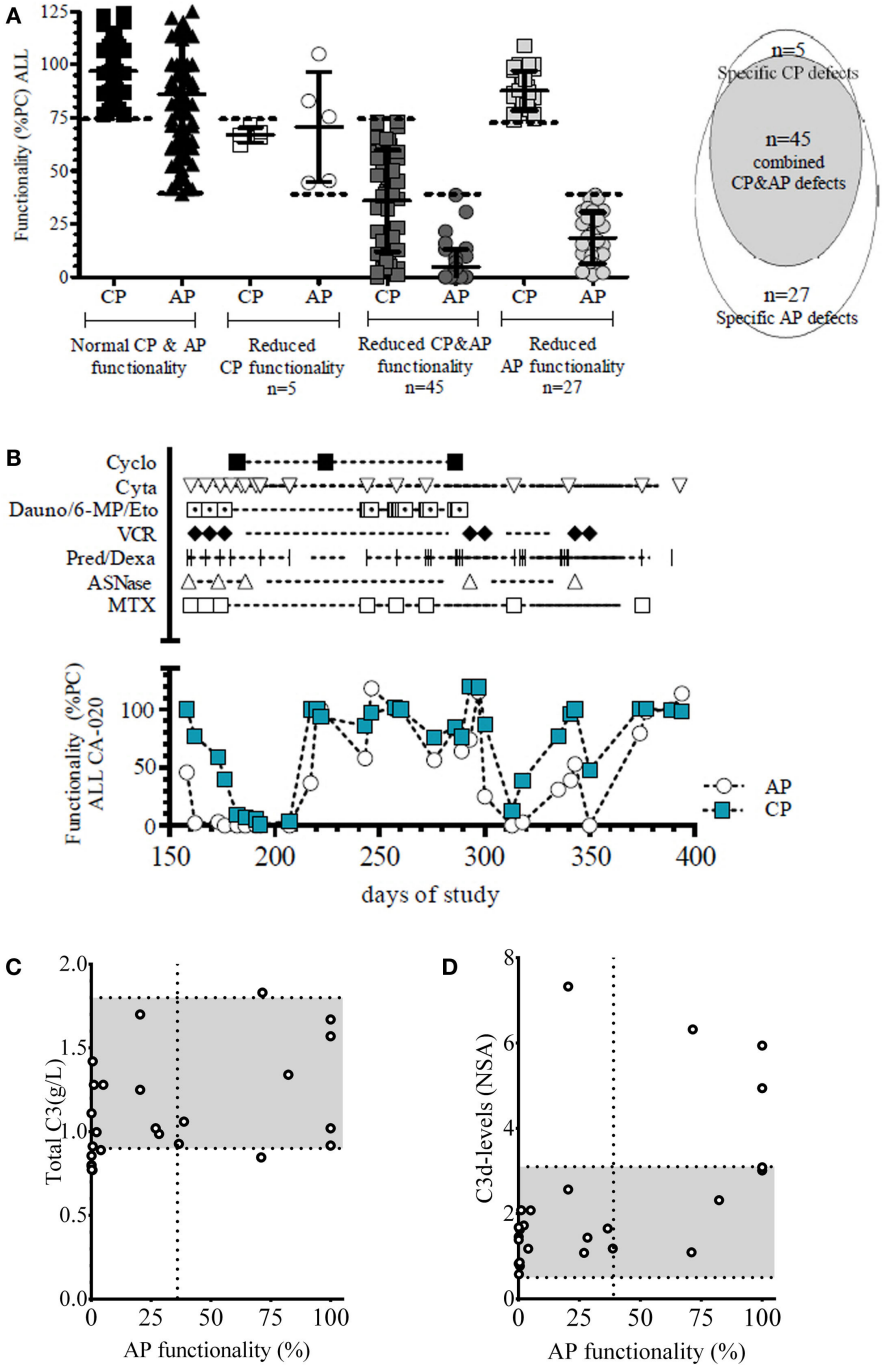
**Temporal effect of ALL-11 protocol on complement functionality**. **(A)** Functionality of the complement system of all patients treated according to the ALL-11 (*n* = 5). The dotted line depicts the lower limit of functionality for CP functionality (<75%) and AP functionality (<40%) as determined for the WIELISA assay. In total, 77 time points showed a reduction in complement functionality in these ALL patients: 5 time points specific for the CP activity, 27 specific for the AP, and 45 specific for both the CP and AP activities. Functionality is depicted as percentage of a positive control (%PC). **(B)** Representative figure of one patient with ALL showing the different treatment compounds with the respective effect on the functionality of the complement system in a longitudinal setting (Cyclo: cyclofosfamide, Cyta: cytarabine, Dauno/6-MP/Eto: Daunorubicin/6-mercaptopurine/etoposide, VCR: vincristine, Pred/Dexa: prednisone/dexamethasone, ASNase: asparaginase, MTX: methotrexate). Functionality is depicted as percentage of a positive control (%PC). **(C)** Total C3 was measured and correlated to the AP functionality. Normal range for total C3 levels (0.9–1.8 g/L) is depicted in gray. **(D)** Complement activation product C3d was measured and correlated to AP activity. C3d levels are compared to fully activated normal serum aged for 1 week (NSA), normal ranges of C3d levels (0.5–3.1) are depicted in gray.

Of the patients with clear abnormalities in the complement activation routes, we analyzed the six largest treatment regimens. Analysis of the different time points (*n* = 200) of the included ALL patients [*n* = 5, all treated according to the ALL-11 protocol (31)] showed a clear negative effect of chemotherapy on the complement pathway activity. We observed only few reductions in complement activity of the CP (*n* = 5/77; 6.5%), compared to the AP (*n* = 27/77; 35.1%), whereas the majority of samples showed simultaneous reductions in both CP and AP activity (*n* = 45/77; 58.4%) (Figure 5A).

Because of the high prevalence of the more common presence of combined defects in both the CP and AP in 60% of the ALL-11 samples, a shared factor was suspected. Temporal analysis of the complement functionality in the individual patients was unable to distinguish a direct correlation to a therapeutic compound (Figure 5B). Upon analysis of total C3 levels to determine possible reductions in protein synthesis or increases in complement consumption, we found neither any correlation with the detected defects in complement functionality (Figure 5C) nor with the levels of C3d, which can be used as a marker for complement activation and consumption (Figure 5D). The ratio between C3d and C3 can be used as a measure of C3 activation (32). No correlation was observed between the ratio of C3d and total C3 and functionality of the CP or the functionality of the AP (data not shown).

**Figure 6:**
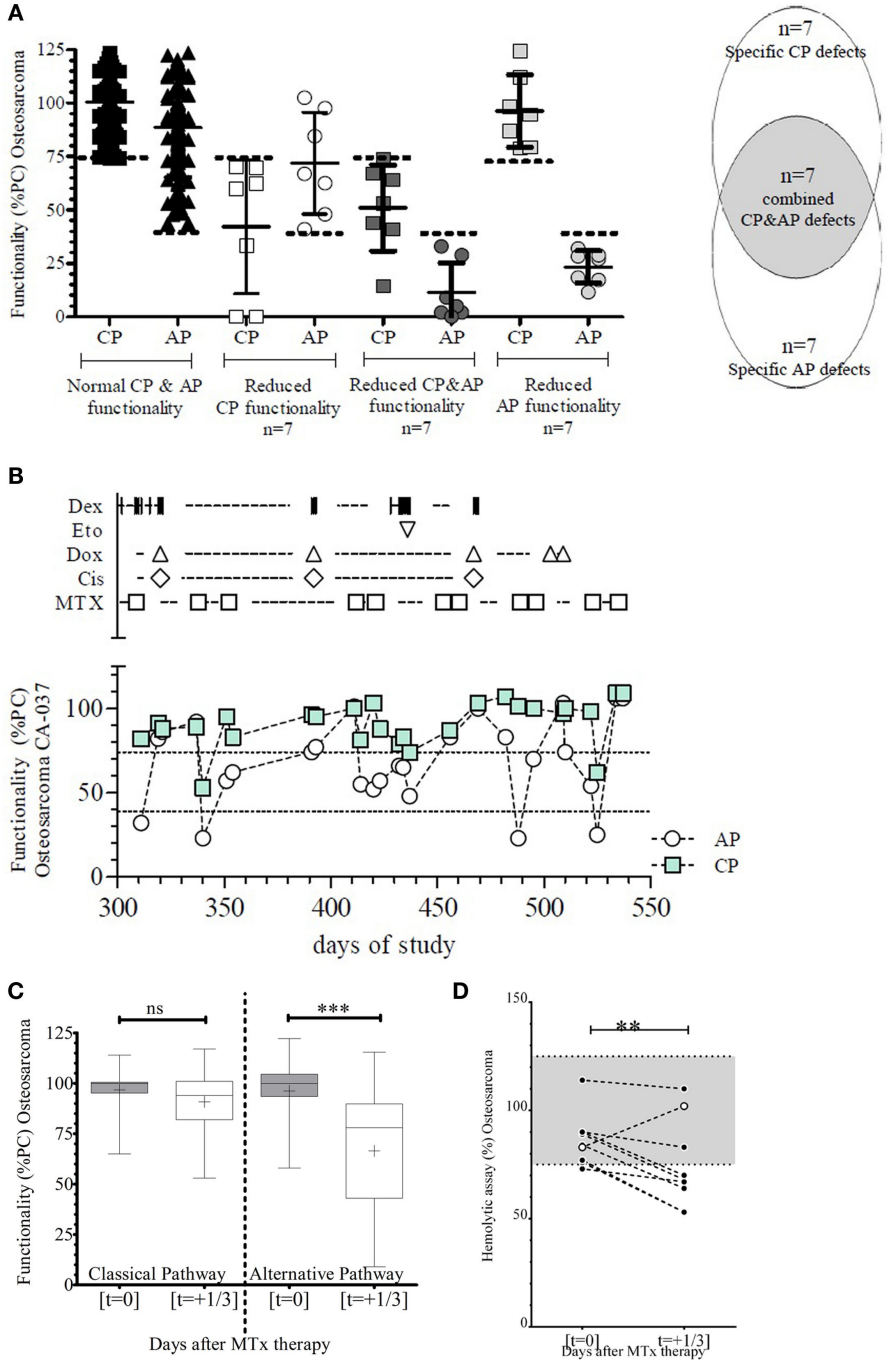
**Classical (CP) and alternative (AP) pathway complement functionality of patients with osteosarcoma receiving high-dose methotrexate (MTX) treatment**. **(A)** Functionality of the complement system of all patients (*n* = 7) with osteosarcoma showed few specific CP defects (*n* = 7) with a similar number of specific AP defects or defects in both pathways. The dotted line depicts the lower limit of functionality for CP functionality (<75%) and AP functionality (<40%) as determined for the WIELISA assay. Functionality is depicted as percentage of a positive control (%PC). **(B)** Representative figure of one patient with osteosarcoma showing the different treatment compounds with the respective effect on the functionality of the complement system in a longitudinal setting (Dex: cyclofosfamide, Eto: etoposide, Dox: doxorubicin, Cis: cisplatin, MTX: methotrexate). Functionality is depicted as percentage of a positive control (%PC). **(C)** Combined time points (*n* = 21) from all patients within the EURAMOS I protocol receiving high-dose MTX therapy. The CP was not affected before or after MTX, whereas the AP showed a significant decrease in functionality (***p < 0.001). Box whiskers depict the min–max values; mean is indicated by a plus symbol. Functionality is depicted as percentage of a positive control (%PC). **(D)** The activity of the alternative pathway measured by the ability to induce hemolysis of sheep erythrocytes (AP50) was significantly decreased following high-dose MTX therapy (**p < 0.01). Dotted lines represent the upper and lower limit of normal reference values. One patient (depicted in open symbol) showed an increase after MTX therapy and subsequent rescue-therapy with Rescuvolin™.

In the subgroup of osteosarcoma patients (*n* = 7) treated according to the EURAMOS I protocol (33), a total of 189 time points were available for measuring the CP and AP. No correlation explaining the specific reduction in complement functionality of the CP or the reduced functionality of the CP and AP was detected (Figure 6A). On the other hand, patients receiving high-dose methotrexate (HD-MTX, 12 g/m^2^) (Table 3) showed less AP functionality directly following MTX infusion [most clearly by combining the different time points (*n* = 21)] (Figure 6B), before and after HD-MTX of all osteosarcoma patients, which showed a significant and immediate decrease (*p* < 0.001) (Figure 6C). To confirm these effects in a different method, AP activity was also tested in a hemolytic assay (AP50), demonstrating a similar decrease in AP functionality after infusion of MTX (*p* < 0.01) (Figure 6D). One pair of plasma samples was excluded (open symbol) because the patient received rescue-therapy (Rescuvolin™) for toxic MTX levels above the upper cutoff used (i.e., 10 μg/L after 24 h; blood levels were closely monitored as part of the protocol). This latter patient showed rapid recovery of AP functionality following rescue-therapy, supporting the direct effect of HD-MTX on the functionality of the AP.

**Table 3:** **Overview of the most commonly used chemotherapeutic compounds in the different treatment protocols**.

Malignancy	ALL	Osteosarcoma	Ewing sarcoma		Rhabdomyosarcoma	Germ cell tumor	Medullablastoma	
Therapy protocol	ALL-11 (31)	EURAMOS I (33)	Ewing 1999 (33)	Ewing 2008 (34)	EpSSG–RMS (35)	SIOP CNS GCT (36)	ACNS 0331 (37)	ACNS 0332 (38)
Actinomycin D			+	**+**	+			
Adriamycin		+			+			
Asparaginase	+							
Busulfan/melphalan			+	+				
Carboplatin						+		+
Cisplatin		+		+	+		+	+
Cyclophosphamide	+							
Cytosine arabinoside	+							
Daunorubicin	+							
Doxorubicin	+		+	+				
Etoposide	+	+	+	+		+		
Fludarabine	+							
Ifosfamide		+	+	+	+	+		
Lomustine							+	
6-Mercaptopurine	+							
Methotrexate	+	+						
Treosulfan/melphalan				+				
Vincristine	+		+	+	+		+	+
Vinorelbine					+			
Zoledronate				+				

Patients with Ewing sarcoma (*n* = 5) received a therapeutic drug regimen of which several compounds were shared with the other treatment subgroups with exception of the alkylating antineoplastic agents busulfan, melphalan, or treosulfan (33, 39) (Table 3). We observed a reduction in the functionality of the CP (*n* = 7/62; 11.3%), whereas a reduction in the AP activity or a combination of the AP and CP activities was negligible and only present once (*n* = 1/62; 1.6%). The treatment protocol for Ewing sarcoma is the only chemotherapeutic treatment protocol, which appeared to be associated with a specific reduction in the CP. Despite all efforts, we were unable to find a direct correlation between these shared or unique drugs and the complement activity measured in the functional assays.

**Figure 7:**
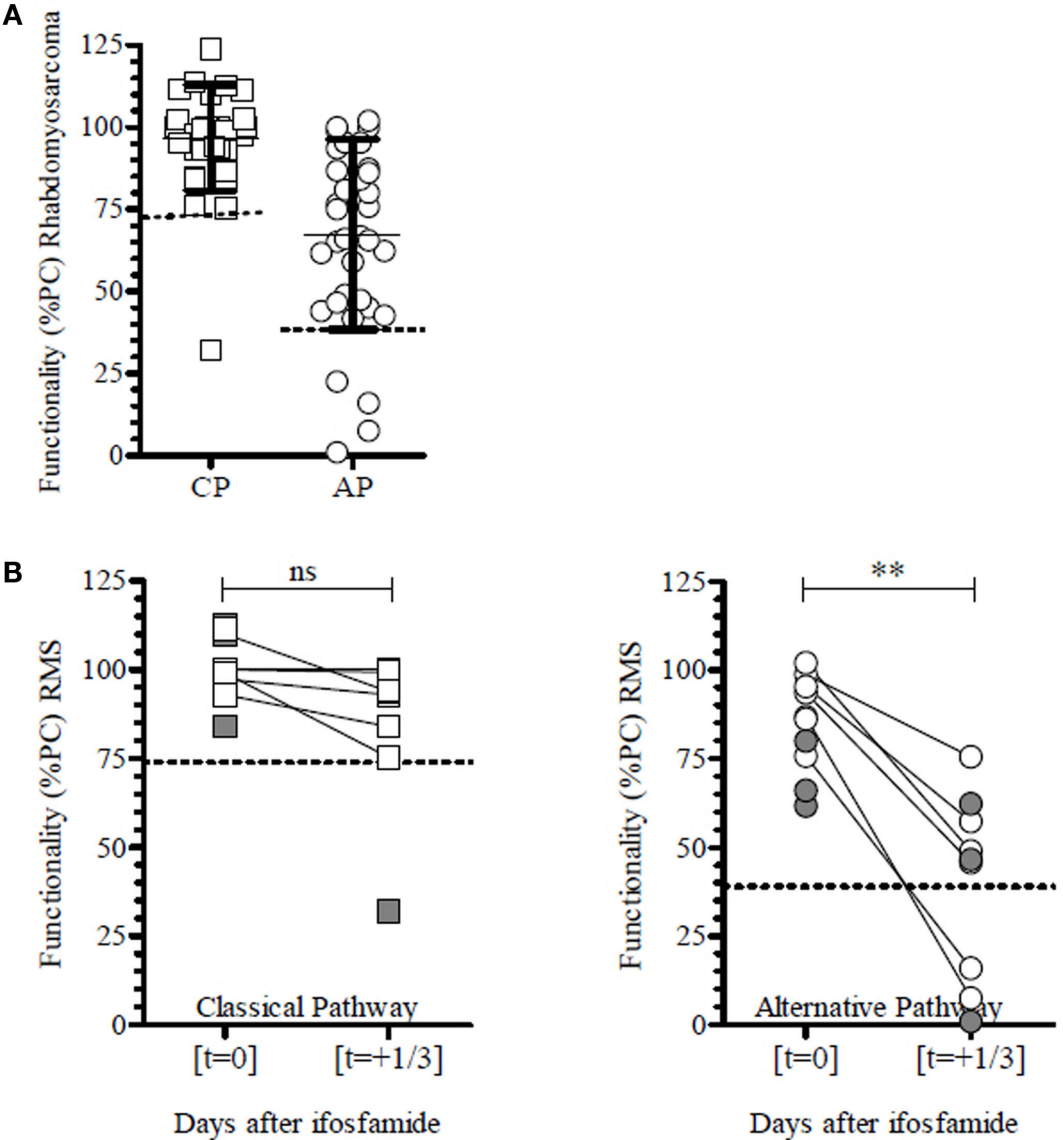
**Classical (CP) and alternative (AP) pathway complement functionality of patients with rhabdomyosarcoma (RMS)**. **(A)** Functionality of the complement system of all patients (*n* = 3) with rhabdomyosarcoma show no specific CP defects, whereas there are few specific AP defects (*n* = 3) and a single combined defect. **(B)** Combined time points from all patients with rhabdomyosarcoma receiving ifosfamide. Although no reduced functionality was observed at all time points, the AP was significantly reduced following therapy, whereas the CP remained unaffected. Unpaired samples are depicted in gray. Dotted lines represent the lower cutoff of normal complement functionality. **p < 0.01; paired *t*-test (*n* = 7). Functionality is depicted as percentage of a positive control (%PC).

Although patients with rhabdomyosarcoma received similar forms of chemotherapy as the patients with Ewing sarcoma (35, 40), only some time points showed a clear reduction in the AP complement activity (Figure 7A). Further analysis indicated that these reductions in AP activity were associated with ifosfamide (Figure 7B). Ifosfamide is also given to patients with osteosarcoma, Ewing sarcoma, or germ cell tumors, but the drug-related effect on the AP activity as observed in rhabdomyosarcoma was not seen in these latter two groups. On the other hand, *post hoc* analysis of time points of the osteosarcoma patients receiving ifosfamide confirmed this drug-related association (Appendix D in Supplementary Material). No correlation of a complement defect with the dosage or other clinical parameters was found. Patients with a germ cell tumor were treated according to the SIOP CNS GCT protocol, which combines radiotherapy and chemotherapy (41, 42), but no specific relation with complement functionality was identified. The small number of patients and/or the limited longitudinal time series that were available in some of the patient subgroups make it difficult to establish a common cause for these transient reductions in complement functionality.

Patients with medulloblastoma (*n* = 3) were treated according to the ACNS-protocol (37, 38), and all patients showed reduced AP activity at certain time points, while they kept an intact functionality of the CP (Figure 4F). In our *post hoc* analysis, we were unable to determine a possible correlation to therapy, neither chemotherapy nor radiotherapy, or other clinical parameters.

## Discussion

Significant reductions in the activating pathways of the complement system were observed in oncology patients. We reproduced our previous observation, showing the reductions of complement activity following chemotherapy were transient of nature (15). In this clinical prospective study, some of oncological treatment regimens were found to specifically affect one or more of the three different activating pathways of the complement system.

In 66% of the patients included, a defect in the CP and/or AP route was observed. A reduced functionality in both pathways was observed in 42.6% of the patients (*n* = 20/47) and in 23.4% of the patients (i.e., 4.3% in the CP and 19.1% in the AP) in a single pathway. Because of the variability of MBL levels in the blood samples (and only one completely MBL-deficient patient), the current analysis focused on the CP and AP pathways of complement.

In these 28 affected patients within the 6 different therapeutic groups, a total of 25% of the time points analyzed showed a reduced function of 1 or more of the activation pathways of the complement system. A reduced CP and AP activity was observed in 14 and 20% of all time points measured. Although there was considerable overlap between CP and AP defects, we identified a number of specific defects, suggesting the presence of as yet unidentified highly selective factors that may impact on these functional complement pathways.

In subsequent *post hoc* analysis, specific chemotherapeutic drugs could be implicated in the inhibitory effect on the complement activity in different patient subgroups, as suggested for leukemia, osteosarcoma, or CNS tumors. The effect of chemotherapy complement components has been previously tested in patients with breast cancer receiving epirubicin–docetaxel combination therapy (18).

As observed in our study, reduced functionality of the complement system could occur by inhibition, reduced production, or by increased turnover and depletion of complement proteins. There are few studies pointing to a selective activation of the classical, lectin, and AP of complement in the presence of different malignancies (43, 44). Cancer cells are known to develop strategies to inhibit and evade complement activation on the membrane by the overexpression of regulatory proteins (45, 46). Systemic inhibition of the complement system has not been described to date. However, if any of the malignant processes *per se* were directly responsible for the systemic complement defects observed, the reduced functionality of the complement system would have been already more prominently present before the start of chemotherapy. Subsequent effective therapeutic sessions would reduce the tumor burden and hereby reduce the inhibitory capacity of the cancer. Similarly, an increased and prolonged consumption, because of tumor cell death as induced by the chemotherapy, would have resulted in a more prominent inhibitory effect on the complement system in earlier therapeutic sessions. Reduction of the tumor load would reduce the amount of consumption of complement components. If an increased consumption could result in a reduced capacity to activate the activation pathway, this would not directly explain a selective inhibitory activation of different pathways depending on the therapeutic regimens. The direct effect of cancer therapeutics on complement components could only be demonstrated for the therapeutic compounds MTX and ifosfamide.

The strong correlation with specific treatment regimens suggests either a direct effect of the drugs as part of the chemotherapeutic regimens on the functionality of (specific) complement proteins or an indirect effect, either *via* the increased cell death of (malignant) cells and depletion of complement components as a consequence, or *via* the reduced production of complement proteins. We were unable to show an increase in consumption of C3 by increased levels of the split product C3d or a decreased protein level of C3 due to increased complement turnover or breakdown. This was also shown by the lack of correlation of the ratio of C3d and C3 and the functionality of the CP or the functionality of the AP. This underlines that the observed transient complement defects are neither related to increased consumption due to cell death nor to an inhibitory effect of the treatment on complement protein synthesis. These findings suggest that, downstream of C3, other late complement components of the common so-called late terminal complement pathway are involved in the observed combined CP and AP defects.

This study was sufficiently powered to determine the presence of transient reductions in complement activity in oncology patients. However, the study was not specifically designed to determine a direct effect of specific therapies or the many combination of chemotherapeutic compounds on the complement system. Nonetheless, we were able to show a correlation between MTX and ifosfamide and their direct negative effect on the complement system.

Due to chemotherapy-related neutropenia, the oncology patients are more dependent on their innate non-cellular immunity for the defense against pathogens. Reduction of complement activity could increase the rate of infection. Oncologic treatment is associated with a higher incidence of infection (47, 48), and this has been previously correlated to levels of complement proteins of different activation pathways (49–51). Due to the relative small number of patients included in the study and the heterogeneous composition of the patient population, a possible clinical association between infection and reduced complement functionality was not feasible. In this study, the number of patients studied and the low number of proven microbial infections were too small to draw any conclusion but having demonstrated the high prevalence of acquired complement defects, prospective studies designed to investigate the potential link between complement and infection-risk has become a feasible next step. However, although reduced functionality of the complement system could be a risk factor for infection, it has been suggested that low-grade inflammation by deposition of sub-lytic amounts could be beneficial, by inducing specific signaling pathways for tumor survival and growth (52), and inhibition of complement could therefore be of therapeutic value for overall survival of the patient.

In summary, oncology patients frequently show transient complement activation defects. Certain chemotherapeutic drugs appear to be directly related to the reduction of the activity of one or more complement pathways. Now that we have established highly significant complement defects in some of the oncology treatment regimens, additional studies should be designed to determine the positive or negative effects of the complement system (antitumor and/or increased risk of infection), which may be more relevant in children being more dependent on the innate immune system than in adults, which may have preexisting specific antibodies being largely absent in young oncology patients.

## Author Contributions

MK was involved in work conception, clinical study design, clinical data retrieval, clinical data analysis, experimental design, experimental work, experimental data analysis, manuscript writing and revision, and final manuscript approval. AK, CA, and JG were involved in experimental design, experimental work, experimental data analysis, manuscript revision, and final manuscript approval. HC was involved in clinical study design, clinical data retrieval, manuscript revision, and final manuscript approval. MW, DW, and TK were involved in work conception, clinical study design, clinical data retrieval and analysis, experimental design, experimental data analysis, manuscript writing and revision, and final manuscript approval. All authors agree for work accountability.

## Conflict of Interest Statement

The authors declare that the research was conducted in the absence of any commercial or financial relationships that could be construed as a potential conflict of interest.
