# Supplementary material for: The High Prevalence of Functional Complement Defects Induced by Chemotherapy
Source: Front Immunol. 2016 Oct 17;7:420. doi: 10.3389/fimmu.2016.00420 (PMC5066094; doi:10.3389/fimmu.2016.00420)
Supplement: Supplementary file 1 [file data_sheet_1.pdf]

Appendix A. **Power analysis of complement study (CCMO registered NL39747.018.12)**

Using the results from our previous MBL-substitution trial in pediatric oncology patients in Amsterdam (15), and data from the international literature, we determined the following number of patients needed for inclusion to answer the primary outcome regarding the presence of transient reduction in complement functionality.

MBL-deficiency has been described in 10 to 18% of the normal population – being largely of Caucasian background(25). In our earlier MBL-substitution trial a transient defect in the alternative pathway was observed in 6 of the 20 episodes in 12 MBL-deficient patients.

For calculation purposes a two-sided significance of 0.05 was used. Determining the level of a transient defect in the complement activity of the AP at 30% or lower of normal baseline activity levels, the difference is 70% (or 0.3 or less) compared to normal healthy donors. Using a standard deviation of 10% (or 0.1) and the formula of Schoenfeld[2], the number of patients for inclusion would be 24 patients.

The activation of the alternative pathway and classical pathway is independent of the activation of the lectin pathway, however the observed effects on the reduced functionality were only seen in MBL-deficient patients. Correcting for the distribution within the normal population of MBL deficiency the number of patients to be required is calculated at a total of 160 patients.

---

[2] http://hedwig.mgh.harvard.edu/sample_size/js/js_associative_quant.html

Appendix B. **Fluctuations of MBL serum levels in our oncology patient cohort**

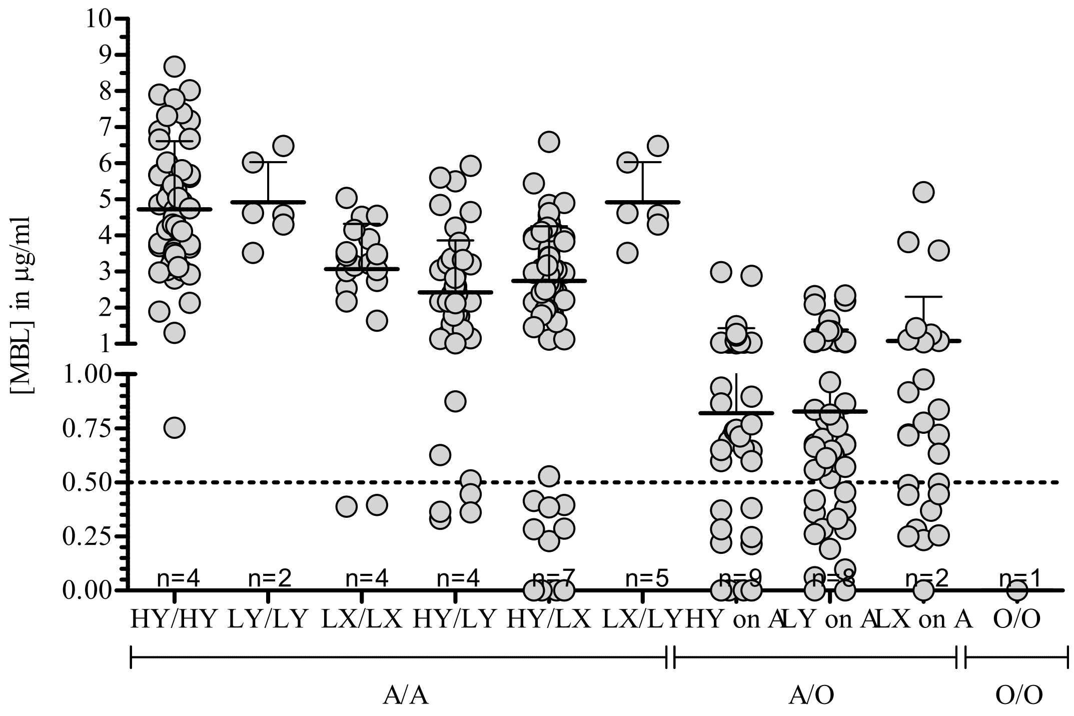

MBL serum levels within the different patients in reference towards their MBL genotype. MBL serum levels are strongly influenced by genotype but are also influenced by an acute phase reaction.

Appendix C. **Overview of complement defects in our oncology patient cohort**

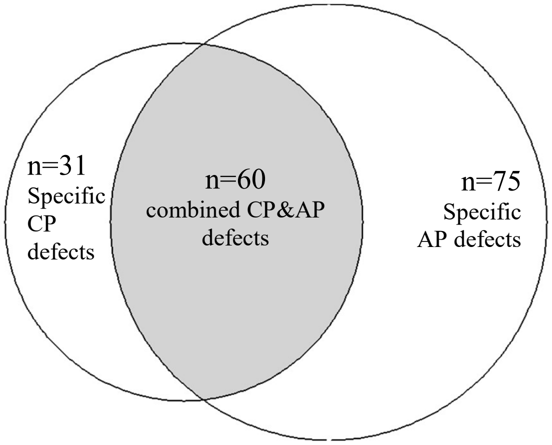

	Therapy protocol	Time points	Complement defects	Specific CP defects	CP & AP defects	Specific AP defects
All patients		**669**	**166 (24.8%)**	**31 (4.6%)**	**60 (9.0%)**	**75 (11.2%)**
	Data of therapy-groups compared to the total groups.					
	ALL-11 n=5	200 (29.9%)	79 (47.6%)[3]	5 (16.1%)[4]	45 (75%)	29 (38.7%)
	EURAMOS I n=7	189 (28.2%)	23 (13.8%)	7 (22.6%)	7 (11.7%)	7 (9.3%)
	Ewing n=5	62 (9.3%)	9 (5.4%)	7 (22.6%)	1 (1.7%)	1 (1.3%)
	EpSSG-RMS n=3	35 (5.2%)	4 (2.4%)	0 (0%)	1 (1.7%)	3 (3.9%)
	SIOP CNS GCT n=3	20 (3.0%)	1 (0.6%)	0 (0%)	0 (0%)	1 (1.3%)
	ACNS n=3	31 (4.6%)	13 (7.8%)	0 (0%)	0 (0%)	13 (7.3%)
	**Total**	537 (80.3%)	129 (77.7%)	17 (54.8%)	56 (93.3%)	56 (74.7%)

[3] 79 complement defects of a total of 166 observed (=47.6%)
[4] 5 specific classical pathway defects on a total of 31 specific classical pathway defects observed (=16.1%)

Appendix D. **Effect of ifosfamide on complement functionality.**

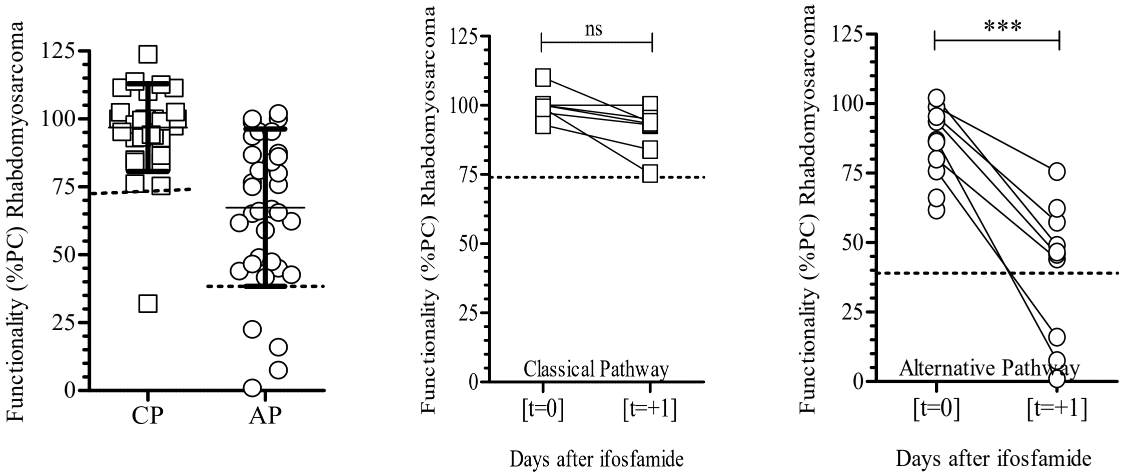

A.

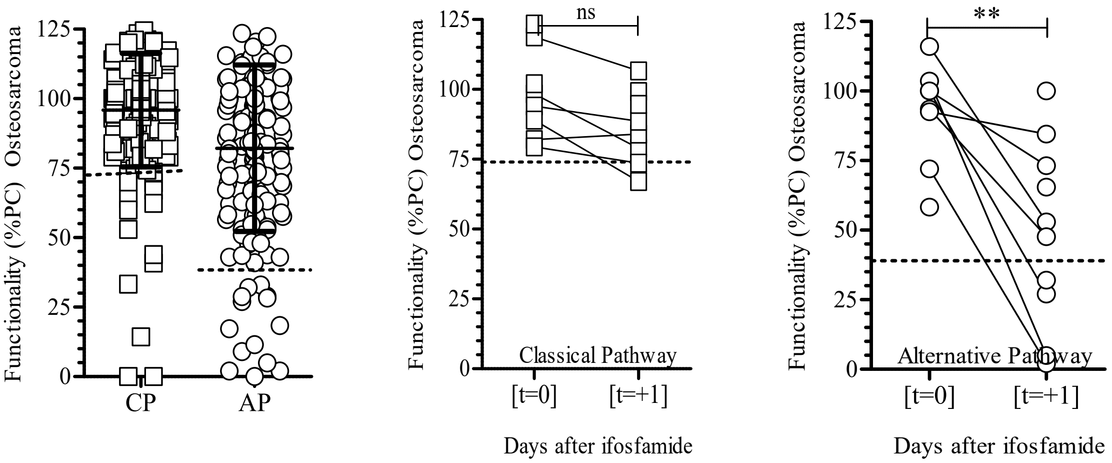

B.

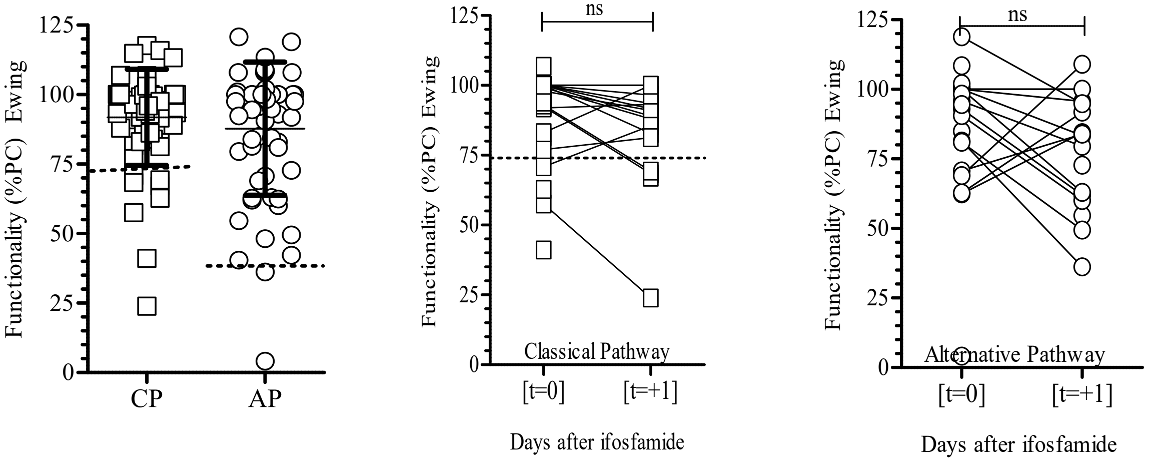

C.

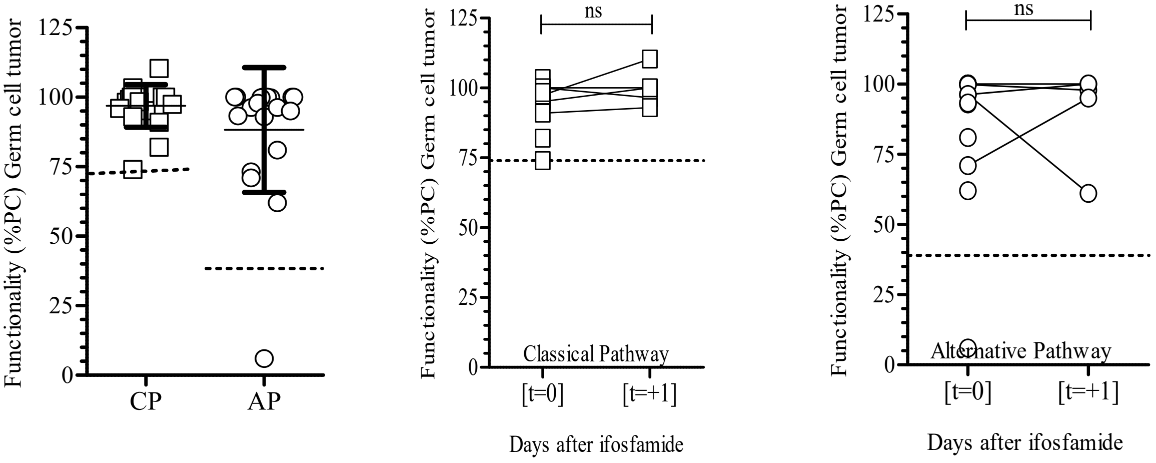

D.

Patients with Rhabdomyosarcoma (A), osteosarcoma (B), Ewing sarcoma (C) or germ cell tumor (D) received ifosfamide as part of their treatment regimens. Where both groups of patients with RMS and osteosarcoma show a reduced functionality of the alternative pathway, this effect is not seen in patients with Ewing sarcoma or germ cell tumor. Dotted lines srepresent the lower cut-off of normal complement functionality. (** p<0.01; *** p<0.001, paired t-test). Functionality is depicted as percentage of a positive controle (%PC).
